# Differentiated, Promoter-specific Response of [4Fe-4S] NsrR DNA Binding to Reaction with Nitric Oxide*

**DOI:** 10.1074/jbc.M115.693192

**Published:** 2016-02-17

**Authors:** Jason C. Crack, Dimitri A. Svistunenko, John Munnoch, Andrew J. Thomson, Matthew I. Hutchings, Nick E. Le Brun

**Affiliations:** From the ‡ Centre for Molecular and Structural Biochemistry, School of Chemistry, and; the ¶ School of Biological Sciences, University of East Anglia, Norwich Research Park, Norwich NR4 7TJ and; the § School of Biological Sciences, University of Essex, Wivenhoe Park, Colchester CO4 3SQ, United Kingdom

**Keywords:** DNA-protein interaction, iron, iron-sulfur protein, nitric oxide, spectroscopy, DNA-binding protein, regulator

## Abstract

NsrR is an iron-sulfur cluster protein that regulates the nitric oxide (NO) stress response of many bacteria. NsrR from *Streptomyces coelicolor* regulates its own expression and that of only two other genes, *hmpA1* and *hmpA2*, which encode HmpA enzymes predicted to detoxify NO. NsrR binds promoter DNA with high affinity only when coordinating a [4Fe-4S] cluster. Here we show that reaction of [4Fe-4S] NsrR with NO affects DNA binding differently depending on the gene promoter. Binding to the *hmpA2* promoter was abolished at ∼2 NO per cluster, although for the *hmpA1* and *nsrR* promoters, ∼4 and ∼8 NO molecules, respectively, were required to abolish DNA binding. Spectroscopic and kinetic studies of the NO reaction revealed a rapid, multi-phase, non-concerted process involving up to 8–10 NO molecules per cluster, leading to the formation of several iron-nitrosyl species. A distinct intermediate was observed at ∼2 NO per cluster, along with two further intermediates at ∼4 and ∼6 NO. The NsrR nitrosylation reaction was not significantly affected by DNA binding. These results show that NsrR regulates different promoters in response to different concentrations of NO. Spectroscopic evidence indicates that this is achieved by different NO-FeS complexes.

## Introduction

The gaseous, lipophilic molecule nitric oxide (NO) is an important signaling molecule in animals and there is growing evidence that it also has a signaling role in bacteria (1). At higher concentrations (micromolar) NO is a cytotoxin, a property exploited by the innate immune response of eukaryotes to infection by pathogenic organisms. The toxicity of NO is conferred by its reactivity toward DNA (nitrosative DNA damage (2)) and proteins (*e.g. S*-nitrosation (3) and *N*-nitrosation (4)) and protein metal cofactors, such as iron-sulfur (FeS) clusters (5), which are important for many cellular functions (6). The generation of NO in the presence of superoxide can also lead to the formation of peroxynitrite, leading to toxic effects (7).

The toxicity of NO is exploited by mammalian macrophages in their response to infection by pathogenic bacteria (8). The ability to sense and respond to high concentrations of NO is therefore a key component of stress response mechanisms of pathogenic organisms (9). Detoxification of NO is also important in many non-pathogenic organisms (10). For example, NO can be generated endogenously at significant concentrations in bacterial cells that are respiring anaerobically using nitrate/nitrite as terminal electron acceptors (11, 12) and NO is generated via the activity of NO synthases in some Gram-positive soil bacteria (13).

NsrR has been identified as a regulator of the NO stress response in a number of bacteria, including *Escherichia coli* (14) *Bacillus subtilis* (15) and pathogens such as *Neisseria gonorrhoeae* (16). In most of the organisms investigated to date, NsrR is a global regulator, controlling a complex network of genes, only some of which are directly related to NO detoxification. In the soil bacterium *Streptomyces coelicolor*, however, NsrR has a more specialized function, regulating only the *nsrR* gene itself and two *hmp* genes (*hmpA1* and *hmpA2*) (24). These genes encode NO detoxifying flavohemeoglobins (17) that convert NO to nitrate (or nitrous oxide under anaerobic conditions). Therefore, in this organism, NsrR appears to regulate only the detection and detoxification of NO.

NsrR is a member of the Rrf2 family of transcriptional regulators, which includes IscR that regulates FeS cluster biosynthesis (18, 19). Like IscR, NsrR contains three conserved cysteine residues in the C terminus region that act as ligands to an iron-sulfur cluster (20–22). Recently it was shown that NsrR from *S. coelicolor* (*Sc*NsrR),^2^ previously reported to contain a [2Fe-2S] cluster (23), can also accommodate a [4Fe-4S] cluster, and that this form alone exhibits high affinity DNA binding to *Sc*NsrR-regulated genes (24), consistent with *Sc*NsrR functioning as a repressor. Furthermore, some non-physiological low molecular weight thiols were shown to promote, in the presence of O_2_, conversion to a [2Fe-2S] form, likely accounting for the [2Fe-2S] form previously reported.

Here we report studies of the effects of NO on DNA binding by [4Fe-4S] *Sc*NsrR along with spectroscopic and kinetic studies of the cluster reaction with NO. The data reveal that DNA binding is abolished at different stoichiometric ratios of NO to cluster, depending on the promoter sequence. Binding of *Sc*NsrR to the *hmpA2* gene promoter was found to be the most sensitive, with binding abolished at ∼2 NO per cluster. Spectroscopic studies revealed a distinct intermediate at the same NO:cluster ratio.

## Experimental Procedures

### 

#### 

##### Purification of S. coelicolor NsrR

Wild type and C terminally His-tagged *Sc*NsrR were purified as previously described (24, 25). Protein concentrations were determined using the method of Smith *et al*. (Pierce) (26) with bovine serum albumin as the standard. Cluster content was determined using an extinction coefficient of ϵ_406 nm_ = 13.30 (±0.19) mm^−1^ cm^−1^ (24).

##### Analytical Methods

Stock solutions of the NO donor PROLI-NONOate (*t*_½_ = 1.5 s; Cayman Chemicals) were prepared in 25 mm NaOH, quantified optically (ϵ_252 nm_ 8400 m^−1^ cm^−1^) and calibrated as previously described (27). For kinetic experiments, an aliquot of PROLI-NONOate was combined with assay buffer (20 mm Tris, 20 mm MES, 100 mm NaCl, 20 mm Bistris propane, 5% glycerol, pH 8.0) and allowed to decompose in a gas tight syringe (Hamilton) to achieve the desired NO concentration before addition to *Sc*NsrR samples.

##### Electrophoretic Mobility Shift Assays (EMSAs)

DNA fragments carrying the *hmpA1* (SCO7428), *hmpA2* (SCO7094), or *nsrR* (SCO7427) promoters were PCR amplified using *S. coelicolor* genomic DNA and band shift assays carried out as previously described (24), but with [4Fe-4S] NsrR following reaction with increasing concentrations of NO.

##### Spectroscopy

For reactions with NO, initial experiments resulted in the observation of a white precipitate in the solution at ratios of NO:[4Fe-4S] of >2. We found that the inclusion of glutathione (0.3 mm) in the buffer solution stabilized the solution against precipitation, even at high levels of NO. Therefore, all spectroscopic studies described here were performed in the presence of glutathione unless otherwise indicated.

UV-visible absorbance measurements were made with a Jasco V500 spectrometer and CD spectra were measured with a Jasco J810 spectropolarimeter. CD titrations were repeated in the presence of a 23-bp double stranded oligonucleotide (dsDNA) that included the *hmp1A* binding site. The dsDNA was annealed from two single strands of DNA (5′-AACACGAATATCATCTACCAAT-3′ and complement strand) according to the manufacturer's instructions (Integrated DNA Technologies). DNA was quantitated via *A*_260 nm_ and the molecular weight for the dsDNA calculated using OligoCalc (28). CD data were noisier than in the absence of DNA, reflecting difficulties associated with working with viscous solutions of DNA (29). Fluorescence measurements were made using an anaerobic fluorescence cell (1-cm path length) in a PerkinElmer LS55 spectrometer.

EPR spectra were recorded on a Bruker EMX (X-band) EPR spectrometer equipped with an Oxford Instruments liquid helium system and a spherical high-quality ER 4122 (SP 9703) Bruker resonator. Composite EPR spectra were deconvoluted into individual EPR signals by using the procedure of spectra subtraction with variable coefficients (30, 31). The concentrations of the paramagnetic complexes in the samples were determined by relating double integrals of the protein EPR spectrum to that of 1 mm Cu^2+^ in 10 mm EDTA standard, both measured at identical instrumental conditions and in the absence of microwave power saturation, *i.e.* at 77 K and P_MW_ = 0.2 milliwatt. EPR spectra simulation was performed by using WinEPR SimFonia version 1.26 (Bruker Analytik GmbH).

EPR samples (250 μl) were prepared by combining aliquots of protein and PROLI-NONOate to achieve the desired [NO]:[FeS] ratio. Samples were incubated at ambient anaerobic glovebox temperature (∼21 °C) for 5 min prior to loading into the EPR tube and freezing.

##### Rapid Reaction Kinetics

UV-visible stopped-flow experiments were performed with a Pro-Data upgraded Applied Photophysics Bio-Sequential DX.17 MV spectrophotometer, with a 1-cm path length cell. Absorption changes were detected at a single wavelength (360 or 420 nm), as previously described (32, 33). Prior to use, the stopped-flow system was flushed with ∼30 ml of anaerobic assay buffer and experiments were carried out using gas tight syringes (Hamilton). All solutions used for stopped-flow experiments were stored and manipulated inside an anaerobic cabinet (Belle Technology). Rapid kinetic experiments were done in the absence of glutathione because precipitation did not occur in the time window of experiments (∼10 s). Fitting of the overall multi-phase kinetic data at 360 and 420 nm (separately and together) was performed using Dynafit (BioKin, CA) (34), which employs numerical integration of simultaneous first-order differential equations, and verified by fitting individual phases to single or double exponential functions using Origin (version 8, Origin Labs). Where appropriate, apparent second order rate constants were obtained from plots of observed rate constants (*k*_obs_) against initial NO concentrations.

## Results

### 

#### 

##### Reaction of [4Fe-4S] NsrR with NO Abolishes Binding to NsrR-regulated Promoters at Different Ratios of NO to Cluster

**FIGURE 1.**
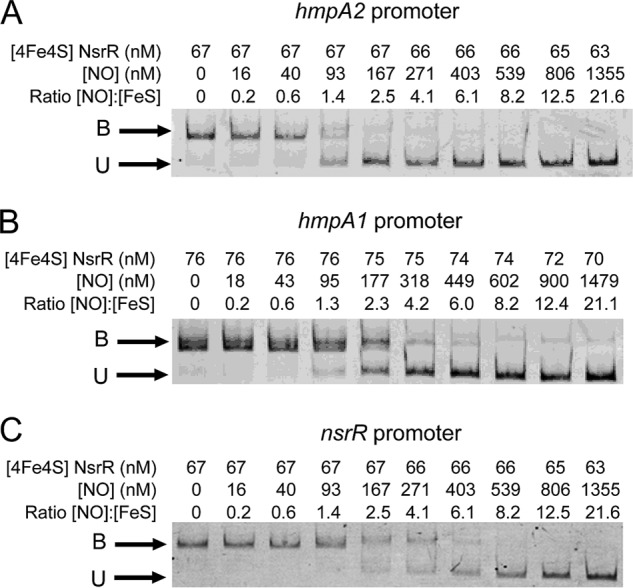
**Effect of NO on *Sc*NsrR DNA binding to NsrR-regulated promoters.** *A*, titration of DNA probe (10.6 nm) containing the *hmpA1* promoter with [4Fe-4S] NsrR following reaction with increasing concentrations of NO, as indicated. *B*, as in *A*, except that the DNA probe (5.9 nm) contained the *hmpA2* promoter. *C*, as in *A* except that the DNA probe (4.6 nm) contained the *nsrR* promoter. The binding buffer contained 10 mm Tris, 54 mm KCl, 0.3% (v/v) glycerol, 1.32 mm GSH, pH 7.5.

It was recently demonstrated that [4Fe-4S] NsrR binds tightly at an 11-bp inverted repeat sequence in the promoters of *hmpA1* and *hmpA2*, in addition to its own promoter (24). Loss of the cluster to form apoprotein, or conversion to form a [2Fe-2S] form, resulted in loss of DNA binding. Because *Sc*NsrR is an NO sensing regulator that controls only three genes, it was of interest to investigate the effect of NO on the DNA binding properties of the [4Fe-4S] NsrR with the *hmpA1*, *hmpA2*, and *nsrR* promoters. EMSA experiments were conducted with fluorescently (6-FAM)-labeled PCR fragments carrying the promoters, [4Fe-4S] NsrR, and increasing concentrations of NO (see Fig. 1). Prior to the addition of NO, full binding of the promoter DNA was observed (24) and addition of NO resulted in gradual appearance of unbound DNA. Binding of NsrR to *hmpA2* was reduced to 50% at a ∼1.4 NO per cluster and was lost entirely by 2.5 NO per cluster (Fig. 1*A*). For *hmpA1* equivalent ratios were ∼2.3 (50% binding) and 4.2 (complete loss of binding) (Fig. 1*B*) and for *nsrR* they were ∼4.1 (50% binding) and 8.2 (complete loss of binding) (Fig. 1*C*). These data demonstrate that DNA binding is abolished at different ratios of NO:[4Fe-4S], depending on the promoter, and that, for the *hmpA2* promoter, DNA binding is entirely lost at ∼2 NO per cluster.

##### The Reaction of [4Fe-4S] NsrR with NO: a Multi-NO Reaction with an Intermediate at 2 NO per Cluster

**FIGURE 2.**
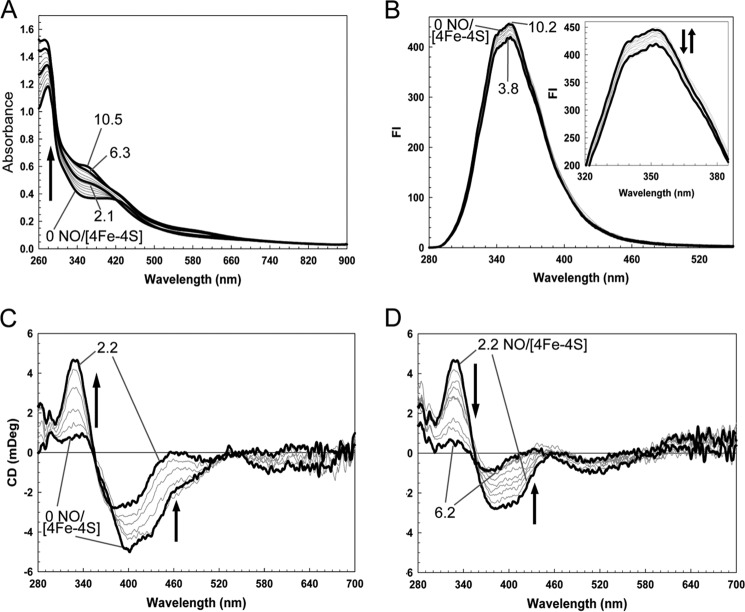
**Titrations of [4Fe-4S] *Sc*NsrR with NO.** *A*, absorbance spectra of [4Fe-4S] NsrR following sequential additions of NO up to a [NO]:[FeS] ratio of 10.5 (*black lines* show spectra recorded at ratios of 0, 2.1, 6.3, and 10.5). *B*, fluorescence spectra obtained during a titration equivalent to that in *A*; *inset* shows changes in more detail. *Black lines* show spectra recorded at [NO]:[FeS] ratios of 0 (*lower*) and 3.8 (*upper*). *C* and *D*, CD spectra obtained during a titration equivalent to that in *A. Black lines* show spectra recorded at [NO]:[FeS] ratios of 0 and 2.2 in *C*, and 2.2 and 6.2 in *D. Arrows* indicate the direction of intensity changes. *Sc*NsrR (28 μm) was in 20 mm Tris, 20 mm MES, 20 mm Bistris propane, 100 mm NaCl, 250 μm GSH, 5% (v/v) glycerol, pH 8.0.

**FIGURE 3.**
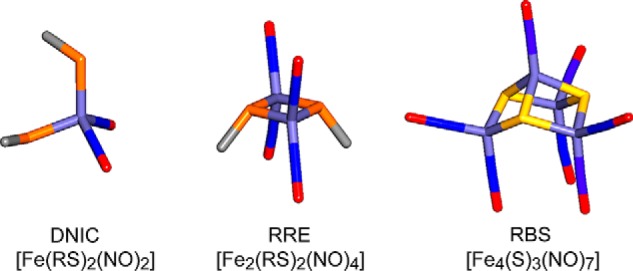
**Iron-nitrosyl species that may be formed following nitrosylation of protein-bound FeS clusters.** Structures of DNIC, RRE, and Roussin's black salt (*RBS*) species are illustrated. Thiolate (RS) groups are shown in *orange*, iron in *light blue*, nitrogen in *dark blue*, oxygen in *red*, and sulfide in *yellow*.

**FIGURE 4.**
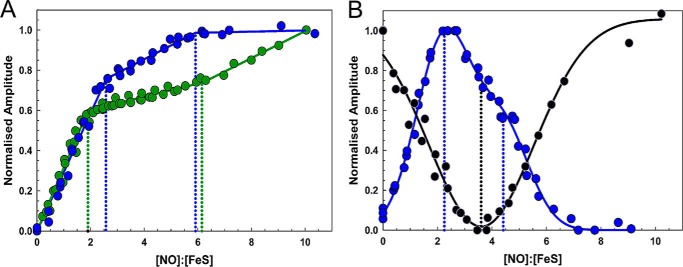
**Plots of spectroscopic changes as a function of NO concentration.** *A*, normalized *A*_360 nm_ − *A*_420 nm_ (*green circles*) and CD_430 nm_ (*blue circles*), and *B*, normalized CD_330 nm_ (*blue circles*), and FI_350 nm_ (*black circles*) plotted *versus* the [NO]:[FeS] ratio. Data are from two independent titrations (data for one of these are shown in Fig. 1).

The reaction of [4Fe-4S] NsrR with NO was investigated by measuring changes in the cluster absorption bands following sequential additions of NO under anaerobic conditions (see Fig. 2*A*). Initial increases in intensity were observed at *A*_360 nm_, and to a lesser extent in the 500–600 nm region. As the titration progressed, further increases in the 360-nm region were observed as the spectrum changed form. The final spectrum, with principal absorption at ∼360 nm and a shoulder at ∼430 nm, is consistent with the formation of iron-nitrosyl species, and closely resembles the spectra of the products formed upon nitrosylation of *S. coelicolor* WhiD and *E. coli* FNR. These were assigned to Roussin's red ester (RRE)-like species (see Fig. 3) (32, 33). RRE complexes exhibit a principal absorption band at 362 nm and a shoulder at ∼430 nm (27, 35). Importantly, no isosbestic points were observed, suggesting a complex reaction pathway involving several intermediates, as illustrated by the highlighted spectra in Fig. 2*A*, which show the form of the iron nitrosyl species changes during the titration. A plot of *A*_360 nm_ − *A*_420 nm_ *versus* [NO]:[4Fe-4S] (Fig. 4*A*) shows that the reaction was complete at a stoichiometry of 8–10 NO molecules per cluster, as observed for other NO-sensitive FeS regulators (32, 33). However, for NsrR there is a clear break point in the plot at a stoichiometry of ∼2 NO molecules per cluster and a further, less distinct one at ∼6 NO per cluster.

Similar changes induced by NO additions were followed by tryptophan fluorescence (FI_353 nm_) (see Fig. 2*B*). The [4Fe-4S]^2+^ cluster acts as a quencher of protein fluorescence (32) but as NO was added, the fluorescence intensity decreased, indicating that the iron-nitrosyl species formed is a more efficient quencher of fluorescence intensity. As further NO was added, intensity recovered to approximately the starting point, indicating the conversion of the initial iron-nitrosyl species (an intermediate) into a different iron-nitrosyl species (product(s)). A plot of fluorescence intensity changes at 353 nm against the ratio [NO]:[4Fe-4S] (Fig. 4*B*) showed the reaction is complete at 8–10 NO, with the formation of the fluorescence detectable intermediate at 3–4 NO per cluster.

The CD spectra in the near UV-visible region of the FeS cluster arise from the chirality imposed by the protein fold. Hence changes in the CD spectra allow reactions with NO to be followed. Sequential NO addition showed major changes during the course of the titration, reflecting formation of intermediates (see Fig. 2, *C* and *D*). The starting spectrum contained a small positive feature at 330 nm and a major negative feature at 400 nm, as previously reported (24). As NO was added, the intensity of the band at +330 nm increased significantly, whereas the band at −400 nm decreased in intensity and shifted to −380 nm (Fig. 2*C*). As further NO was added, the +330 nm band was lost and the remaining intensity at −380 nm decreased and shifted further to −370 nm. A broad negative feature was also observed at 520 nm (Fig. 2*D*). A plot of CD intensity at 430 nm (Fig. 4*A*) showed that changes were complete at ∼6 NO per cluster, with a clear break at ∼2 NO. An equivalent plot of CD intensity changes at 330 nm (Fig. 4*B*) very clearly showed the formation of an intermediate at 2 NO per cluster, which subsequently reacts with further NO to give a less distinct intermediate at ∼4 NO with the CD response essentially complete at 7–8 NO. All three forms of UV-visible spectroscopy absorption, fluorescence, and CD data show a complex reaction course with [4Fe-4S] NsrR clearly forming intermediates at ∼2 NO, and ∼4 and ∼6 NO molecules, with no further reaction beyond 8–10 NO per cluster.

##### Reaction with NO Is Not Significantly Affected by [4Fe-4S] NsrR DNA Binding

**FIGURE 5.**
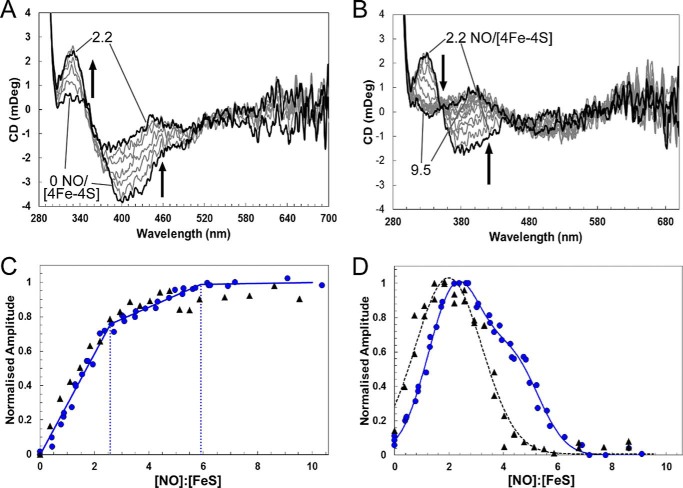
**The effect of DNA binding on [4Fe-4S] *Sc*NsrR reaction with NO.** CD spectra of [4Fe-4S] NsrR (16 μm [4Fe-4S] NsrR dimer, 32 μm [4Fe-4S]) following sequential additions of NO. *A* and *B* show CD spectra obtained during a titration equivalent to that in Fig. 2, *C* and *D,* in the presence of 32 μm dsDNA. *Black lines* show spectra recorded at [NO]:[FeS] ratios of 0 and 2.2 in *A*, and 2.2 and 9.5 in *B. Arrows* indicate the direction of intensity changes. *Sc*NsrR was in 10 mm Tris, 54 mm KCl, 0.3% (v/v) glycerol, 1.5 mm GSH, pH 7.5. *C* and *D*, *black triangles* show normalized CD intensity at 430 and 330 nm, respectively, plotted *versus* the [NO]:[FeS] ratio for reaction in the presence of DNA. Equivalent data for reaction in the absence of DNA is replotted (*blue circles*) from Fig. 3 for comparison. Data are from two independent titrations (data for one are shown in *A* and *B*).

Because NsrR is a regulatory protein, it will encounter NO when bound to DNA. It was therefore of interest to determine whether the DNA binding affects the reaction of [4Fe-4S] NsrR with NO. CD was used to investigate this because, of the spectroscopic methods above, it gave the most distinctive response to the NO reaction. Thus a CD titration was repeated with [4Fe-4S] NsrR bound to a 23-mer oligomer containing the NsrR-binding sequence of the *hmpA1* promoter, previously found to bind NsrR with highest affinity of all NsrR-regulated promoters (24). In the presence of DNA, the response of the negative feature at −400 nm was essentially identical to that observed in the non-DNA bound form (Fig. 5, *A* and *B*), with breaks in the response at ∼2 and ∼6 NO per cluster (Fig. 5*C*). The intermediate species detected at +330 nm was also observed to form and decay in a similar way. Maximum intensity occurred at ∼2 NO per cluster, slightly shifted compared with the absence of DNA (Fig. 5*D*). Some differences were observed at higher ratios of NO, such that the shoulder observed at ∼4 NO in the absence of DNA was not detected in its presence (Fig. 5*D*), but this may be due to the increased noise of the spectra. Overall, the major features of the NO responses are similar for [4Fe-4S] NsrR free in solution or bound to DNA.

##### EPR Spectroscopy of Nitrosylated [4Fe-4S] NsrR Reveals the Formation of DNIC Species

**FIGURE 6.**
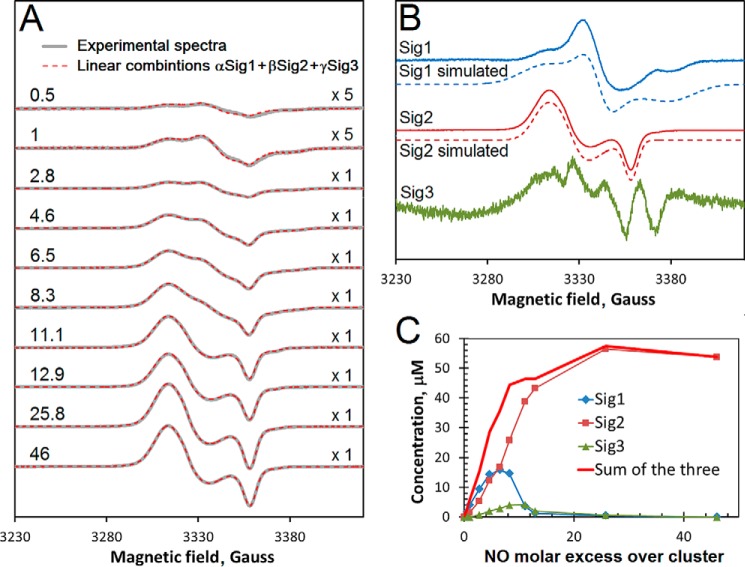
**EPR analysis of DNIC formation during reaction of [4Fe-4S] NsrR with NO.** *A*, EPR spectra following the addition of NO to 100 μm [4Fe-4S] NsrR (*gray lines*). The spectrum of NsrR prior to NO treatment was subtracted from each. The [NO]:[FeS] ratios are indicated. The first two spectra are magnified by a factor of 5 indicated by the “x” symbol (on the *right*). The experimental data are overlaid with linear combinations of the three EPR signals shown in *B*. The coefficients α, β, and γ used in these linear combinations are given in Table 1. *B*, the three EPR signals (*solid lines*) assumed to be basic components of all spectra shown in *A*, were obtained as described under ”Experimental Procedures.“ Signals 1 and 2 were simulated (*dashed lines*) with the following parameters: Sig1, *g_x_* = 2.0440, *g_y_* = 2.0246, and *g_z_* = 2.0000 (Δ*H_x_* = 25 G, Δ*H_y_* = 12 G, Δ*H_z_* = 25 G); Sig2, *g_x_* = 2.0426, *g_y_* = 2.0332, and *g_z_* = 2.0140 (Δ*H_x_* = 14 G, Δ*H_y_* = 14 G, Δ*H_z_* = 7 G). *C*, concentrations of the species responsible for EPR signals Sig1, Sig2, and Sig3 as functions of the excess of NO over cluster. Spectra were recorded at 77 K. Microwave power and frequency were 3.18 milliwatts and 9.47 GHz, respectively, and field modulation amplitude was 0.3 millitesla. The sample buffer was 50 mm Tris, 2 m NaCl, 5% (v/v) glycerol, pH 8.0.

Reactions of protein-bound FeS clusters with NO were first observed by EPR spectroscopy, through the detection of paramagnetic mononuclear iron dinitrosyl (DNIC) species (36, 37) (Fig. 3). This provides a means of quantifying the amount of DNIC species formed during the course of reactions with NO (32, 33, 38, 39). Therefore, EPR spectroscopy was used to assess the formation of DNIC species upon nitrosylation of [4Fe-4S] NsrR. NO was added to NsrR in increasing NO:[4Fe-4S] ratios from substoichiometric to large excess, allowed to react for 5 min and then frozen for EPR measurements. Prior to the addition of NO, the spectrum was devoid of signals, consistent with the presence of diamagnetic [4Fe-4S]^2+^. On addition of NO signals in the *g* = ∼2 region, characteristic of the S = ½ DNIC species, were observed increasing in intensity with increasing ratio of NO to cluster (see Fig. 6*A*). Analysis of the spectra revealed that each can be deconvoluted into three distinct signals that contribute to different extents to the evolving spectra (see Fig. 6*B* and Table 1). Signal 1 (Sig1) was simulated as a S = ½ species with *g_x_* = 2.0440, *g_y_* = 2.0246, and *g_z_* = 2.0000, and signal 2 (Sig2) as a S = ½ species with *g_x_* = 2.0426, *g_y_* = 2.0332, and *g_z_* = 2.0140. Signal 3 (Sig3) could not be simulated as a single species and even at its maximum was of very low intensity in the observed spectra. Up to a ratio of ∼6 NO per cluster, signals 1 and 2 contributed equally to the observed spectrum, but above this ratio, signal 1 decayed away and signal 2 grew further. Signal 2 is characteristic of a Cys-coordinated DNIC, but signal 1 is not similar to previously characterized DNIC species (40) and so may represent another type of iron-nitrosyl species. The relatively small increase in DNIC intensity observed beyond ∼10 NO per cluster most likely results from some conversion of multinuclear iron-nitrosyl species (see later) into DNICs. Spin integration of the signals yielded a total maximum concentration equivalent to ∼60% of the original [4Fe-4S] concentration; that is, ∼15% of the iron originally present as the cluster.

**TABLE 1:** **Coefficients in the linear combinations (αSig1 + βSig2 + γSig3) of signals 1, 2, and 3 (Fig. 6*B*) used for simulations of the experimental spectra shown in Fig. 6*A***

NO excess	α	β	γ
0.5	0.0315	0.0134	0.0000
1	0.0724	0.0291	0.0000
2.8	0.1725	0.0994	0.0106
4.6	0.2650	0.2275	0.0256
6.5	0.2917	0.3120	0.0369
8.3	0.2700	0.4800	0.0513
11.1	0.0650	0.7227	0.0525
12.9	0.0223	0.8034	0.0258
25.8	0.0092	1.0500	0.0075
46	0.0000	1.0000	0.0000

##### [4Fe-4S] NsrR Cluster Reacts Rapidly with NO

**FIGURE 7.**
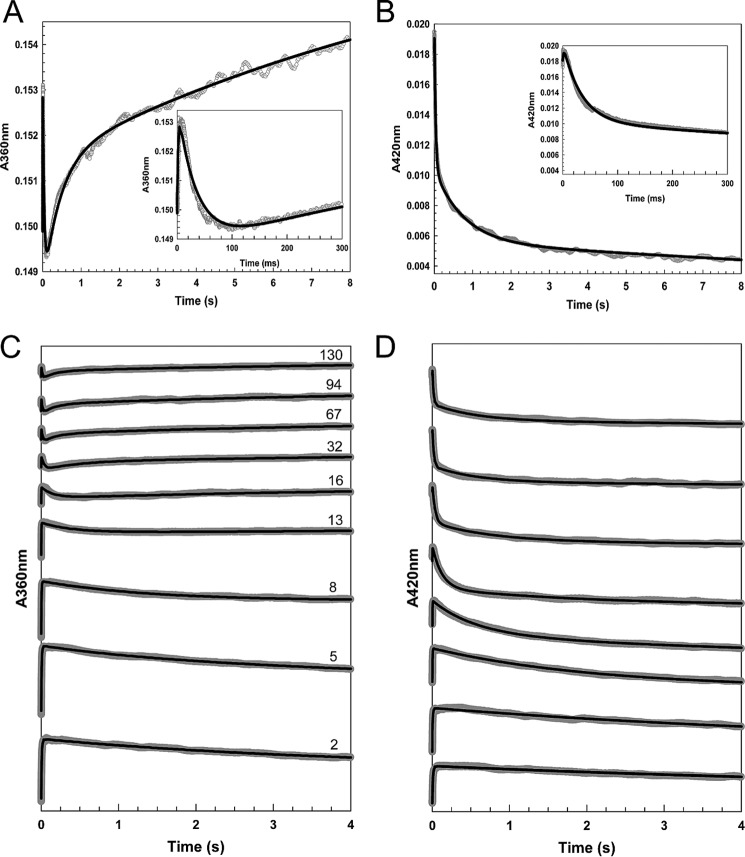
**Stopped-flow measurements of the reaction of [4Fe-4S] NsrR with NO.** *A–D*, absorbance at 360 (*A* and *B*) and 420 nm (*C* and *D*) following the addition of NO to NsrR (∼7.6 μm). *A* and *C* show data at 360 and 420 nm, respectively, for the addition of ∼32 NO molecules per cluster. *B* and *D* show data at 360 and 420 nm, respectively, for a range of other NO:cluster ratios, as indicated. *Insets* in *A* and *C* show early events in the reaction time course. Fits to each of the observed phases (see ”Experimental Procedures“) are drawn in *black lines*.

The reaction of [4Fe-4S] NsrR with excess NO (NO:[4Fe-4S] ∼32) was followed using stopped-flow absorbance spectroscopy, monitoring *A*_360 nm_ and *A*_420 nm_ as a function of time. These wavelengths correspond to the maxima of the final nitrosylated product and the initial iron-sulfur cluster, respectively (Fig. 7). A rapid, multiphase reaction was observed at both wavelengths, as previously observed for other FeS regulators (32, 33). The data were fitted separately, and together, to exponential functions, giving equivalent results. Analysis revealed the presence of four phases at 360 nm and three phases at 420 nm. The first two phases were detected at both wavelengths, but the remaining phases had different kinetic characteristics (at the two wavelengths), indicating that they report on different processes. Thus, overall, the third phase was detected at 360 nm, the fourth at 420 nm, and the final phase at 360 nm. Thus, the overall reaction was modeled as a five step reaction, *i.e.* A → B → C → D → E → F, where the initial (A → B) and second (B → C) steps are detected at both wavelengths, C → D is detected at 360 nm, D → E at 420 nm, and the final step E → F at 360 nm.

**FIGURE 8.**
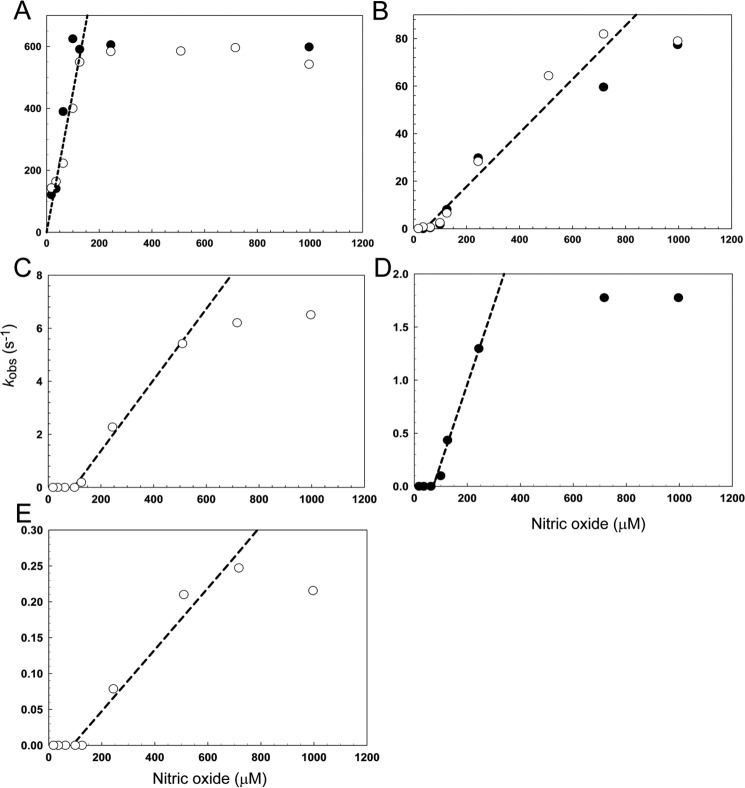
**Dependence of the observed rate constant for each step of nitrosylation on NO.** *A–E,* plots of the observed (pseudo-first order) rate constant (*k*_obs_), obtained from fits of the kinetic data at 360 (*open circles*) and 420 nm (*filled circles*), over a range of NO concentrations. Note that *panels A–E* correspond to steps 1 to 5, respectively, of the reaction (see text). Least squares linear fits are shown giving apparent second order rate constants (see Table 2). The buffer was 20 mm Tris, 20 mm MES, 20 mm Bistris propane, 100 mm NaCl, 5% (v/v) glycerol, pH 8.0.

Experiments were repeated at NO:[4Fe-4S] ratios ranging from 2 to 130 and the data fitted as described under ”Experimental Procedures,“ to give observed rate constants. Plots of observed rate constants (*k*_obs_) against the NO concentration are shown in Fig. 8. The observed rate constant for the first step (A → B) exhibited, initially, a first order dependence on NO (measured at both 360 and 420 nm) (see Fig. 8*A*). The gradient of the dependence gave a second order rate constant of ∼4.5 × 10^6^ m^−1^ s^−1^ (see Table 2). At ∼150 μm NO, the reaction became independent of NO, indicating that at higher NO concentrations, the rate determining step switched to a process that does not involve NO.

**TABLE 2:** **Apparent second order rate constants for the five observed steps in the nitrosylation reaction of [4Fe-4S] *Sc*NsrR and comparison with those of *S. coelicolor* WhiD (32) and *E. coli* FNR (33)** Note that, with the exception of the initial reaction (A → B), the rate constants for NsrR may not represent the same reaction as for WhiD and FNR.

Phase	Step	Rate constant		
		NsrR	WhiD	FNR
		*m*^−*1*^ *s*^−*1*^		
1	A → B	4.52 ± 0.23 × 10^6^	4.40 × 10^5^	2.81 × 10^5^
2	B → C	1.13 ± 0.11 × 10^5^	1.38 × 10^4^	1.89 × 10^4^
3	C → D	1.34 ± 0.10 × 10^4^	8.34 × 10^3^	4.61 × 10^3^
4	D → E	7.48 ± 0.69 × 10^3^	0.90 × 10^3^	0.75 × 10^3^
5	E → F	0.43 ± 0.05 × 10^3^		

Step 2 (B → C) was found to be linearly dependent on NO (Fig. 8*B*), giving a rate constant an order of magnitude lower than that for step 1 (Table 2). Step 3 (C → D) was linear with NO in the range ∼100–500 μm with a rate constant lower again by an order of magnitude (Fig. 8*C*, Table 2). At lower concentrations, insufficient amplitude was detected in the few seconds of measurement for the phase to be fitted. At higher NO, the reaction became independent of NO concentration. Steps 4 (D → E, Fig. 8*D*) and 5 (E → F, Fig. 8*E*) were similar in that they were not detected at low NO but were linearly dependent on NO at intermediate NO concentrations before becoming NO independent at high NO concentrations. The rate constants determined from the linear parts of the plots were sequentially lower by an order of magnitude than that for the previous step (Table 2).

## Discussion

Here we provide novel biochemical insight into why the response to NO may not be uniform for all genes controlled by a single (FeS) regulator. DNA band shift experiments revealed that the response of the three *Sc*NsrR-bound promoters to NO was different, with *hmpA2* the most sensitive and *nsrR* the least sensitive. This implies a hierarchy of expression response to NO. Interestingly, previous studies of [4Fe-4S] NsrR binding to DNA revealed that binding to the *hmpA2* promoter was the weakest (24). However, there is no clear correlation with promoter binding affinity because binding to *hmpA1*, which here was found to have intermediate sensitivity to NO, exhibited the strongest binding to [4Fe-4S] NsrR (24).

Analysis of the reaction of [4Fe-4S] NsrR with NO using spectroscopic and kinetic methods revealed a complex process involving reaction of up to 8–10 NO per cluster, but with a series of intermediates, at ∼2, 4, and 6 NO per cluster, formed along the nitrosylation pathway. Thus the *Sc*NsrR nitrosylation reaction is not concerted, *i.e.* NO does not react preferentially to completion with clusters that have already undergone initial reaction, relative to those that have not yet reacted.

These studies follow related investigations of other NO-sensing FeS regulators, including *Mycobacterium tuberculosis* WhiB1, *S. coelicolor* WhiD, and *E. coli* FNR (32, 33). The data reported here for *Sc*NsrR bear similarities to those regulators, in that reaction involves multiple NO molecules and results in iron-nitrosyl products. However, whereas an intermediate was observed at ∼4 NO:[4Fe-4S] for the reaction of [4Fe-4S] FNR (33) and possibly also [4Fe-4S] WhiD (32), no other intermediates, particularly at low ratios of NO to cluster, have been detected previously.

Importantly the spectroscopic observations link to the DNA binding data. Binding of *Sc*NsrR to the *hmpA2* promoter was entirely abolished at ∼2 NO per cluster, indicating that the *Sc*NsrR intermediate species detected at this ratio can no longer bind *hmpA2* promoter DNA. For *hmpA1* and *nsrR* promoters, ∼4 and ∼8 NO molecules per cluster, respectively, were required to abolish DNA binding. For *hmpA1*, this ratio also corresponds to an intermediate observed via spectroscopy, whereas for *nsrR*, it suggests that the full nitrosylation reaction is needed to abolish binding. Previous studies of cluster reactivity of a DNA-bound FeS regulator revealed that the rate of reaction was affected but the overall mechanism was not (29). The clear correlation between observed intermediates and DNA binding behavior of *Sc*NsrR suggests that it is unlikely that DNA binding significantly affects the mechanism of the NsrR nitrosylation reaction, and this conclusion is consistent with CD measurements showing that DNA-bound *Sc*NsrR reacted with NO similarly to *Sc*NsrR free in solution. Although the structures of the intermediate species formed cannot be determined from the current data, a key conclusion from this work is that complete reaction with the FeS cluster of this NO-sensing regulator is not a requirement and DNA binding can be switched off at low ratios of NO to cluster.

The kinetic data revealed a rapid, complex multiphase reaction that was modeled most simply as a five step reaction. The first step of the reaction with NO (A → B; first order with respect to NO) is very likely the binding of one NO molecule to the cluster. The second step (B → C; first order with respect to NO), generating the intermediate with clear spectroscopic characteristics, results from the binding of a second NO, which could be at the same iron or elsewhere on the cluster. As in previous studies of FeS regulators, it is difficult to assign the identity of this species and those resulting from subsequent steps of the reaction because the form of the iron, and how it changes during the reaction as the cluster breaks down, cannot be determined from these data and are, in fact, extremely difficult to identify unambiguously. Each step shows a linear dependence on NO (at least initially where they are detected), indicating that they correspond to the sequential binding of NO to iron. Individual steps could involve the binding of more than one NO, but this would involve independent binding of NO to different irons of the cluster, giving an overall first order dependence.

The rate constant for the initial step of the *Sc*NsrR NO reaction is an order of magnitude greater than that detected for WhiD from the same organism (Table 2) (32). Furthermore, the rate constant for the slowest step of the NO reaction is at least ∼3 orders of magnitude greater than that for the slowest step of the reaction with O_2_ (24). These observations are consistent with a role for *Sc*NsrR as the first line of defense against NO, *i.e.* from kinetic analyses NsrR would be predicted to preferentially react with NO in the *S. coelicolor* cytoplasm containing both NsrR and WhiD.

The kinetic data also support the conclusion that the *Sc*NsrR cluster nitrosylation is not a concerted reaction. Under conditions of excess NO, five phases were detected, but at low NO:cluster ratios, only the early phases of the reaction were observed, consistent with there being sufficient NO to achieve the formation of only the first intermediates of the full reaction. The effect of this is that plots of observed rate constants *versus* NO concentration have an unusual appearance for the mid/latter phases, in that there are zero values at low NO (Fig. 8).

In the case of WhiD, the cluster is coordinated by four Cys residues, whereas NsrR is coordinated by three Cys residues and one oxygenic residue that previous DNA binding and spectroscopic data suggested might be Glu-85 (24). Previous studies of the effect of low molecular weight thiols suggested that the oxygenic ligand can be readily displaced to generate an all thiol-coordinated cluster (24), and so the unique iron site of the cluster is the most likely site of initial NO binding. The kinetic data showed that the observed rate constant for the initial binding becomes independent of NO above a particular concentration (Fig. 8*A*). At this point, the slow step of the reaction does not involve NO and we propose that, at high NO concentrations, the dissociation of the existing (oxygenic) ligand to the iron, permitting binding of NO, is the rate-limiting step. The rate constant for this process, from the plot in Fig. 8*A*, is estimated to be ∼600 s^−1^.

The form of the final absorbance spectrum of the nitrosylated cluster is similar to those observed previously with WhiD and FNR (32, 33). Although the nature of the iron-nitrosyl species cannot be determined solely from its absorbance properties, these are consistent with RRE-like [Fe^I^_2_(NO)_4_(Cys)_2_] species, as proposed for WhiD and FNR (32, 33), rather than DNIC species, which have distinct absorbance properties (27, 35). However, the spectra could also arise from more complex iron-nitrosyl species such as those related to Roussin's black salt (see Fig. 3) (39). For WhiD and FNR, only minor amounts of DNICs ([Fe^I^(NO)_2_(Cys)_2_]) were detected (<4% total iron) (32, 33). In the case of *Sc*NsrR, significantly more DNIC species, up to 15% of the total iron, was detected by EPR; however, this is still a relatively minor component of the products.

### 

#### 

##### Concluding Remarks

The data presented here reveal novel aspects of NO sensing by an FeS regulatory protein, with distinct responses of DNA binding to NO depending on the sequence of the promoter. Intermediates of cluster nitrosylation, particularly that detected at ∼2 NO per cluster, correlate well with DNA binding behavior, pointing to their physiological importance. Further investigations will be needed to try to establish the precise nature of these intermediates.

## Author Contributions

J. C. C. carried out the bulk of the data acquisition and analyses, helped to design the experiments, and co-wrote the manuscript. D. A. S. performed EPR experiments and analyzed data. J. M. performed some gel shift experiments and assisted with experiments on the effects of DNA binding on the nitrosylation reaction. A. J. T. co-wrote the manuscript. M. I. H. helped conceive and coordinate the study and co-wrote the manuscript. N. L. B. conceived and coordinated the study and wrote the manuscript.

## Footnotes

2 The abbreviations used are:

*Sc*NsrR: *S. coelicolor* NsrR
FeS: iron-sulfur
DNIC: dinitrosyl iron complex
RRE: Roussin's red ester
Bistris propane: 1,3-bis[tris(hydroxymethyl)methylamino]propane.
